# Notes on Surgical Cases

**Published:** 1861-03

**Authors:** E. Andrews

**Affiliations:** Prof. of Surgery in the Medical Department of Lind University


### NOTES OF SURGICAL CASES.

BY E. ANDREWS, M. D.,
Prof. of Surgery in the Medical Department of Lind University.

### *Terrible Neuralgia of the face, cured by excision of a portion of the infra-orbital nerve.*

This case is the same one alluded to in the report of a clinical lecture, given in the Examiner of last month. The patient for several years had been the victim of terrible suffering from neuralgia in all those branches of the superior maxilary nerve which come through the infra-orbital canal. No other twigs being affected, I judged that the foramen was the seat of difficulty. Wishing to use mild measures first, I injected beneath the skin a half grain of morphine in solution, in the manner recommended in certain French publications.

This measure which I have often found surprizingly beneficial, had but a slight influence in this case, and as the patient's life was an intolerable burden in his present condition, I determined to relieve him by excising a portion of the nerve *behind the infra-orbital foramen.*

For this purpose the patient was anæsthetized, and I raised the cheek by dissecting up a semilunar flap as high as the edge of the orbit, exposing the nerve at its exit from the foramen. I then applied the trephine with its upper edge just below the foramen, and taking out a button of bone laid open the antrum. It was now easy to trace the infra-orbital canal backwards by a slight ridge odservable on the roof of the antrum. Commencing at the foramen anteriorly with gouge forceps first, and more delicate instruments as I proceeded backward, I easily broke down the floor of the infra-orbital canal, and traced the nerve back into the cavity of the orbit. Then dislodging it from its groove, I took a pair of small blunt pointed scissors and cut it off as far within the orbit as I could safely reach, taking away about an inch and a quarter of its trunk. The flap was then laid down and secured by sutures, except a small space at the lower edge, which was left open for the escape of pus.

On awaking from the influence of ether, the patient found the character of his pain changed from the shooting neuralgic torture to the steady aching of a surgical injury. This gradually subsided, and by the fourth day he was free from suffering, and on the tenth day was discharged apparently cured.

The want of permanency in the cures obtained by excising portions of nerves, has been heretofore a discouraging feature in such operations. I think, however, that we are now in a condition to understand the matter more thoroughly than before, and to avoid the errors which cause the failure.

A large portion of the operations heretofore have been made in the *wrong places*, namely—on the *distal* instead of the *proximal* side of the seat of the disease. *The seat of disease in old facial neuralgias is generally at some bony notch or foramen through which the nerve passes*, and not at the surfaces of distribution to which the patient refers the sensation. Probably the nerve is pressed upon by some slight constriction of the foramen or swelling of its contents. Now this fact has been greatly overlooked and even the eminent and judicious Prof. Gross, in his new work on surgery, absurdly suggests the cutting of the maxilary nerves *just outside* of their foramina of exit. Other distinguished writers make the same mistake, and many operations have been performed in consequence, at the wrong place. If the excision cannot be made on the proximal side of the seat of disease the operation of course is a failure. Prof. Carnochan, who reports a greater success than any other man, even goes through the back wall of the antrum in neuralgias of the superior maxilary, and takes out Meckel's ganglion. This, however, is probably unnecessary when the point of trouble is in the infra orbital canal, but would be requisite if the palatine nerve were affected.

Finally, there are cases where no useful operation is possible. Those in which the seat of disease is the foramen rotundum or the foramen ovale, of course are incurable by surgical means, because no section can be made within those openings. The diagnosis must be made out by carefully observing the area over which the pain is felt and studying the origin and course of the branches of the 5th nerve supplying the part. If the nerve is affected at a particular point only, all the branches beyond that point will suffer, and all others will be comparatively free from pain. By thus carefully investigating the cases, I am confident that many can be selected in whom operation will effect permanent cures.

